# Comparative Analysis of Lepidopteran Community Structure Using DNA Metabarcoding: Warm‐Temperate Forest Versus Grass‐Shrub Ecotones in Pangquangou National Nature Reserve

**DOI:** 10.1002/ece3.73738

**Published:** 2026-05-27

**Authors:** Ling Wu, Ziyu Huang, Xinrun Ren, Huimin Yao, Min Li, Angie Deng, Yali Li, Shiyi Lian, Lina Men, Zhiwei Zhang

**Affiliations:** ^1^ Shanxi Key Laboratory of Efficient Cultivation of Forest Resources, College of Forestry Shanxi Agricultural University Jinzhong China; ^2^ College of Biological Science and Technology Taiyuan Normal University Jinzhong China; ^3^ Lucile Packard Children's Hospital at Stanford Palo Alto California USA; ^4^ Shanxi Dangerous and Grassland Pest Inspection and Identification Center Jinzhong China

**Keywords:** DNA metabarcoding, insect diversity, Lepidoptera, Pangquangou National Nature Reserve, pest monitoring

## Abstract

Lepidoptera is a widely geographically distributed insect order that plays crucial ecological roles in forest and grass‐shrub ecosystems as both pollinators and herbivores. This study compared Lepidoptera species composition and diversity between semi‐arid forest and grass‐shrub ecosystems in northern China's Pangquangou National Nature Reserve to provide scientific insights for biodiversity conservation and pest management. Nine representative plots (five forest and four grass‐shrub) were established, and Lepidoptera communities were analyzed using an integrated approach combining DNA metabarcoding, DNA barcoding, and classical morphological methods. A total of 2944 individuals were collected, representing 453 species from 80 genera and 31 families, with an 82% cross‐method species identification match. Despite being more time‐consuming, metabarcoding offers a clear advantage for large‐scale biodiversity monitoring due to its extremely low per‐sample cost (up to 79.2% of costs saved compared to traditional morphological methods and 87.3% of costs saved compared to DNA barcoding). However, morphological validation remains necessary to ensure accurate identification. Dominant families showed higher relative abundance in forests (85.07%) than in grass‐shrub areas (75.18%). Alpha diversity, assessed via the Shannon‐Wiener index, exhibited no significant difference between ecosystems (*p* = 0.72). Beta diversity analysis also indicated comparable community compositions, though differences were not statistically significant (*p* = 0.332). Five dominant families (Noctuidae, Notodontidae, Geometridae, Erebidae, and Nymphalidae) were shared between ecosystems, while Lasiocampidae occurred exclusively in forests. Notably, metabarcoding data accurately reflected species composition and abundance, facilitated precise prediction of caterpillar pest outbreaks, and supported targeted control measures. This study confirms that DNA metabarcoding, when supplemented with morphological verification, is a robust tool for Lepidoptera biodiversity assessment. Our findings provide reliable solutions for ecosystem monitoring and pest management, underscoring the method's potential for large‐scale biodiversity studies.

## Graphical abstract

This study confirms that the method based on DNA metabarcoding qualifies as a cost‐efficient alternative to traditional approaches, as well as a better understanding for future assessment of insect diversity.

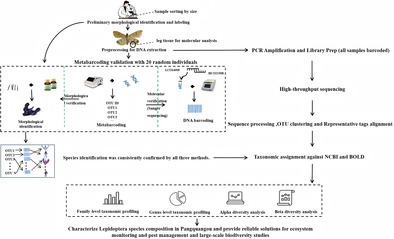

## 1 Introduction

Lepidoptera (moths and butterflies), constituting the second largest insect order (Meng et al. 2019), exhibit remarkable species richness and diversity. While approximately 160,000 species have been formally described worldwide (Brady et al. 2021), current estimates suggest up to 500,000 extant species exist, with around 1000 new species discovered annually (Kristensen et al. 2007). As keystone components of terrestrial ecosystems, Lepidoptera perform multiple ecological functions (Shashank et al. 2022): plant community dynamics are influenced by larval insect herbivory and adult pollination, and both life stages provide essential nutrition for predators, thereby facilitating energy transfer within food webs (Summerville et al. 2004; Hui et al. 2008). Their sensitivity to environmental changes enables Lepidoptera community structure to reflect vegetation succession patterns and signal disturbances at higher trophic levels (Cooke et al. 2025), establishing them as excellent bioindicators for ecosystem health evaluation.

Traditional morphological approaches encounter substantial limitations in Lepidoptera diversity studies (Piper et al. 2019), including taxonomic expertise shortages, numerous under‐described species, and ambiguous larval diagnostic features, all contributing to identification uncertainties (Joern and Laws 2013; Doi et al. 2016). DNA barcoding has emerged as a valuable complementary technique, utilizing standardized short DNA sequences for species identification (Hebert et al. 2006, 2003). The advent of DNA metabarcoding has further transformed this field through high‐throughput sequencing of complex samples (e.g., frass or pupal cases), enabling rapid and comprehensive species composition analyses (Yunzhi et al. 2022). Compared with conventional methods, metabarcoding demonstrates superior cost‐efficiency, high throughput, and operational simplicity, leading to its widespread adoption in ecological investigations ranging from dietary studies (Quanbo et al. 2024) to aquatic biodiversity assessments (Cui et al. 2024) and arthropod community monitoring (Hein et al. 2024).

Our research was conducted in the Pangquangou National Nature Reserve, situated in the central Lvliang Mountains. This representative warm‐temperate montane ecosystem features a distinctive habitat mosaic comprising natural secondary forests and shrub‐grassland ecotones along elevational gradients, supporting exceptional biodiversity (Zhuangzhuang 2018). The region's high‐altitude conditions result in relatively short Lepidoptera activity periods (Gao et al. 2023). Previous studies have predominantly examined butterflies, rove beetles, and ground beetles, creating a significant knowledge gap regarding molecular‐based evaluations of complete Lepidoptera communities, especially comparative analyses across different ecosystems. This limitation obstructs comprehension of biodiversity maintenance mechanisms in transitional zones.

Through light trapping and sweep‐netting across nine discrete plots in two dominant ecosystems (forests versus grass‐shrubs), we collected Lepidoptera specimens for DNA metabarcoding analysis. Our objectives were to characterize Lepidoptera species composition in Pangquangou, compare community differences between forest and grass‐shrub ecosystems, and establish a scientific foundation for biodiversity conservation and ecological management in the reserve.

## 2 Materials and Methods

### 2.1 Sampling

The study was conducted in Pangquangou National Nature Reserve, Shanxi Province, China encompassing geographic coordinates from 111°26′11″ E to 111°31′27″ E and 37°47′33″ N to 37°53′51″ N. Nine study plots were systematically established and classified into two distinct ecosystem types (Table 1).

**TABLE 1:** Basic information on study plots.

Ecosystem type	Plot name	Abbreviation
Forest	Badaogou	BDG
	Xitagou	XTG
	Guanzimao	GZM
	Heiquling	HQL
	Zhugangou	ZGG
Grass‐shrub	Hengjian village	HJV
	Dacaoping	DCP
	Kangdongzi	KDZ
	Bashuigou	BSG

**FIGURE 1:**
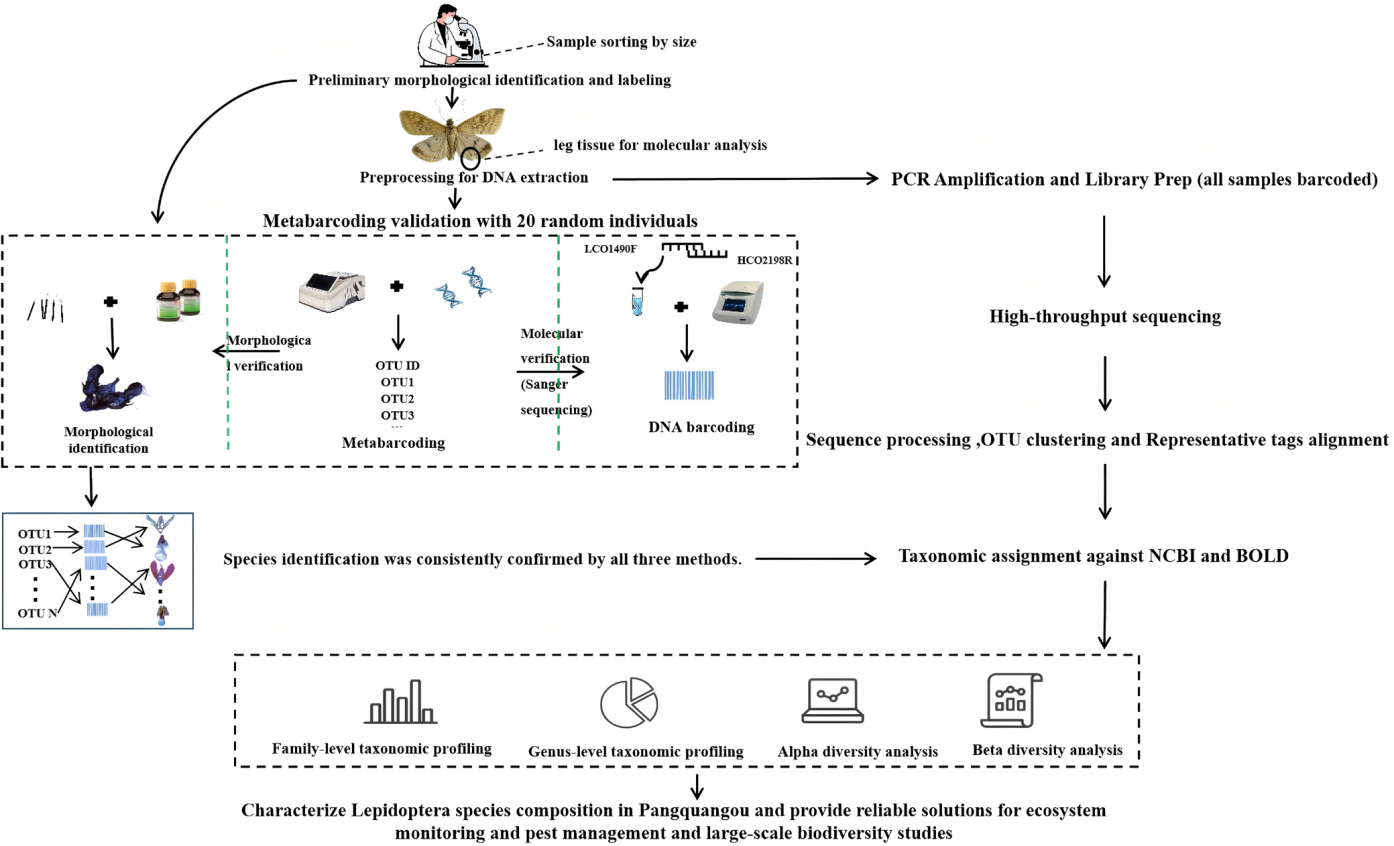
Research methodology framework of this study.

Sampling was conducted from June to August 2023 using sweep netting and light trapping methods. To account for the migratory behavior of Lepidoptera, light traps were positioned within 100 m of the sampling plots, with specimens collected within this radius considered representative of their respective plots. Daytime sampling employed sweep netting within the natural ecosystem, while nocturnal sampling utilized light traps in adjacent flat terrain. All collected specimens were individually preserved in 1.5 mL microcentrifuge tubes containing 99% ethanol, with ethanol replacement performed every 24 h to maintain optimal tissue preservation. Samples were subsequently transported to the laboratory and stored at −20°C in ultralow‐temperature freezers for molecular analyses (Figure 1).

### 2.2 Experimental Methods

#### 2.2.1 Preliminary Morphological Screening and Metabarcoding Pretreatment

Lepidoptera specimens collected from nine sampling plots were first sorted morphologically. To minimize biomass‐related abundance biases prior to pooled DNA extraction, specimens were stratified by size into three categories based on body dimensions. Specimens were classified into small (body size ≤ 2.5 × 5 mm), medium (2.5 × 5 mm < body size ≤ 5 × 10 mm), and large (5 × 10 mm < body size ≤ 10 × 20 mm) size categories. Each size class was flash‐frozen in liquid nitrogen and homogenized through cryogenic grinding. The resulting powders were proportionally recombined based on original mass‐to‐quantity ratios to create composite samples reflecting natural size distributions. Before DNA extraction, leg tissues were dissected and inserted into 1.5 mL centrifuge tubes, washed 3–4 times with deionized water to eliminate ethanol residue and contaminants, then air‐dried at room temperature.

For metabarcoding molecular identification, this study utilized a destructive DNA extraction method targeting leg tissues. The mitochondrial cytochrome coxidase subunit I (COI) gene served as the target DNA fragment. The partial mitochondrial COI gene (313 bp) was amplified as the target region using primers mICOIintF (5′‐GGWACWGGWTGAACWGTWTAYCCYCC‐3′) and jgHCO2198 (5′‐TANACYTCNGGRTGNCCRAARAAYCA‐3′) (Brandon‐Mong et al. 2015). To improve sample representation, each specimen's homogenized tissue was partitioned into multiple subsamples proportional to the total homogenate mass, yielding 2944 homogenized subsamples for DNA extraction. Genomic DNA was extracted from pooled samples using the E.Z.N. A Mag Bind Soil DNA Kit (Omega, M5635‐02, USA) according to the manufacturer's protocol. DNA concentrations were quantified using a Qubit 4.0 Fluorometer (Thermo Fisher Scientific, USA) to verify sufficient yields of high‐quality genomic DNA.

#### 2.2.2 Validation of DNA Metabarcoding Effectiveness Through Conventional Barcoding and Morphological Analyses of Selective Specimens

To ensure representation across body size, samples were randomly selected from the total sample according to the proportions of large‐ (5 × 10 mm < body size ≤ 10 × 20 mm), medium‐ (2.5 × 5 mm < body size ≤ 5 × 10 mm), and small‐bodied (body size ≤ 2.5 × 5 mm) individuals. A total of 29 small‐bodied, 16 medium‐bodied, and 5 large‐bodied specimens were chosen for the systematic assessment of metabarcoding identification accuracy across different body size groups. Samples were transferred to 1.5 mL centrifuge tubes containing distilled water and incubated for 20 min to ensure complete ethanol removal from both external surfaces and internal tissues. After air‐drying on sterile filter paper, leg and head segments were dissected for genomic DNA extraction via cetyltrimethylammonium bromide (CTAB) protocol. DNA quality was verified through UV spectrophotometric analysis and 1% agarose gel electrophoresis. The mitochondrial COI barcode region was amplified with universal insect primers (LCO1490F: GGTCAACAAATCATAAAGATATTGG/HCO2198R: TAAACTTCAGGGTGACCAAAAAATCA) (Folmer et al. 1994). PCR products were confirmed by 1% agarose gel electrophoresis before commercial sequencing by Sangon Biotech Co. Ltd.

Specimens selected for concurrent DNA barcoding and morphological studies were processed as follows. Ethanol‐preserved samples were carefully dissected to isolate abdominal segments using fine forceps. These segments underwent 20 min distilled water immersion for ethanol removal, followed by air‐drying on filter paper. Tissue clearing was achieved through 20 min digestion in 10% NaOH solution. After complete clearance, genitalia were microdissected under stereomicroscopic observation. Genital structures and cuticular features were permanently mounted in Euparal medium on labeled glass slides for comparative morphological studies with reference materials.

### 2.3 Data Analysis

#### 2.3.1 Bioinformatic Processing of Metabarcoding Data

Paired‐end Illumina reads were assembled based on sequence overlap using PEAR (v.0.9.8), with subsequent format conversion from FASTQ to FASTA/QUAL for downstream analyses. Quality‐filtered sequences were clustered into operational taxonomic units (OTUs) at a ≥ 97% similarity threshold using USEARCH software (v.11.0.667), followed by removal of chimeras and singletons (sequences lacking duplicates) Operational Taxonomic Units (OTUs), after which the remaining sequences were sorted into each sample based on the OTUs.

OTU clustering was conducted on quality‐filtered sequence data. For each sample, dereplicated sequences were generated from the processed reads to reduce computational redundancy (Usearch command: fastx_uniques; http://drive5.com/usearch/manual/cmd_fastx_uniques.html). Pooled dereplicated sequences from all samples underwent singleton removal to eliminate potential noise artifacts (http://drive5.com/usearch/manual/singletons.html). Non‐singleton dereplicated sequences were clustered into OTUs at 97% similarity using the UPARSE algorithm, with simultaneous chimera detection and removal (Usearch command: cluster_otus; http://drive5.com/usearch/manual/cmd_cluster_otus.html). Processed reads were mapped to OTU representatives (≥ 97% similarity) to construct an abundance matrix (Usearch command: otutab; http://drive5.com/usearch/manual/pipe_otutab.html). Taxonomic annotation of representative sequences utilized the BOLD Systems database (https://www.boldsystems.org/) for cytochrome coxidase subunit I (COI) gene regions, with locus‐specific databases employed for other genetic markers.

Species‐level assignments required ≥ 97% similarity to BOLD System v4 reference sequences, incorporating associated morphological data. For matches below this threshold, phylogenetic analysis of the top 100 BLAST matches enabled conservative classification at higher taxonomic ranks (genus/family) following Ratnasingham and Hebert (Ratnasingham and Hebert 2007) protocols. All identifications were cross‐validated against the BOLD Systems database (www.boldsystems.org) to ensure taxonomic reliability.

#### 2.3.2 Validation of Metabarcoding Results

##### 2.3.2.1 DNA Barcode Data Processing

Unidirectionally sequenced DNA barcode data were quality‐checked using SnapGene software, with chromatogram visualization and manual trimming of terminal noise peaks. Universal primer regions were excised prior to sequence validation using the “Find Conflicts” error‐checking function. Verified sequences were exported as FASTA files and subjected to comparative analyses against BOLD and NCBI reference databases, retaining matches with ≥ 90% sequence similarity for taxonomic assignment and compared with traditional morphology and metabarcoding.

##### 2.3.2.2 Morphological Validation

Genitalia morphology was examined using stereomicroscopy of dissected slide preparations. Species identifications were corroborated through consultation of authoritative taxonomic literature, including: *Lepidoptera of Southeastern Shanxi Forests* (Bai, 2014), *Henan Insect Fauna: Lepidoptera, Pyraloidea* (Li and Ren, 2009), *Butterflies and Moths of Lushan Mountain* (Fang, 2003), *The Fauna Sinica* monograph series (Chen, 1999), and so on. Peer review by taxonomic specialists ensured morphological consistency between molecular identifications. The integrated approach combined ≥ 90% sequence similarity thresholds with diagnostic morphological characters to validate metabarcoding results.

#### 2.3.3 Statistical and Ecological Analyses

##### 2.3.3.1 Data Filtering and Standardization

We utilized comprehensive reference datasets from both the NCBI NT and the full BOLD Systems repository in order to maximize taxonomic resolution at the genus and species levels (Marrama‐Rakotoarivony and Zeller 2012). For taxonomic annotation, representative sequences from each OTU were analyzed via BLASTn searches against the NCBI NT database (ftp://ftp.ncbi.nih.gov/blast/db/) using default parameters. Only matches demonstrating > 90% sequence similarity and > 90% query coverage were retained for taxonomic assignment (Rees et al. 2014). Following taxonomic classification, all OTUs identified as non‐insect taxa were systematically excluded from subsequent analyses. To standardize sequencing depth across samples, rarefaction was performed using Mothur (v.1.45.3), generating an insect‐specific OTU table (Li et al. 2023). Sampling completeness and sample adequacy were evaluated through rarefaction curves of observed OTUs, which were visualized on the Cloud‐TUTU online platform (https://www.cloudtutu.com.cn) and in R (v.3.6.0).

##### 2.3.3.2 Alpha Diversity Analysis

Community α‐diversity was quantified using five metrics: Chao1 (species richness estimator), Goods coverage (sequencing depth index), Shannon evenness (community uniformity), Shannon and Simpson indices (diversity measures). These metrics were calculated from OTU tables in Mothur software (v.3.8.31).

##### 2.3.3.3 Beta Diversity and Visualization

Inter‐sample β‐diversity was analyzed using Bray‐Curtis and Jaccard dissimilarity matrices, visualized through dimensionality reduction methods including principal coordinate analysis (PCoA) and non‐metric multidimensional scaling (NMDS). These analyses were implemented in the R vegan package (v.2.5‐6). β‐diversity patterns among different ecosystem types were specifically examined via NMDS ordination.

##### 2.3.3.4 Community Composition and Structure Visualization

Venn diagrams illustrating the distribution of insect OTUs across different sample groups were constructed using the Venn Diagram and UpsetR packages in R. Taxonomic abundance patterns at family and genus levels across all sampling sites were visualized through heatmap analyses generated in Hiplot.

## 3 Results

### 3.1 Comparative Analysis of DNA Barcoding, Classical Morphology, and DNA Metabarcoding Results

Analysis of 50 randomly selected insect specimens (Table S1) demonstrated 82% congruence (41/50) across morphological identification, DNA barcoding, and metabarcoding results. Among the remaining specimens, 9 (18%) exhibited inadequate morphological characterization due to insufficient taxonomic documentation, yet both DNA barcoding and metabarcoding consistently identified it as the same species.

### 3.2 Cost‐Effectiveness Comparison of Identification Methods

For the cost analysis of Lepidoptera identification, we evaluated temporal and economic expenditures in three areas (Table 2): current market price of laboratory reagents, commercial DNA barcoding/metabarcoding sequencing fees, and Shanxi Province's 2025 minimum hourly wage for part‐time workers. Calculations were standardized per 50 samples. The analysis revealed that DNA metabarcoding offers a substantial economic advantage, reducing costs by 79.2% compared to morphological methods. Morphological identification required 10 days (total labor cost: 1856 CNY) and relied heavily on taxonomic expertise and extensive literature review. DNA barcoding also took 8 days (total cost including sequencing: 3044.8 CNY). In contrast, metabarcoding cost 385.6 CNY, though its library preparation and bioinformatics analysis required a longer processing time (15 days). Morphological identification of 50 samples required 10 days and cost 1856 CNY. DNA barcoding required 8 days and cost 3044.8 CNY. DNA metabarcoding required 15 days but cost only 385.6 CNY, representing a 79.2% cost reduction compared to morphology and 87.3% compared to barcoding.

**TABLE 2:** Economic and time costs of analyzing 50 Lepidoptera samples using metabarcoding, DNA barcoding, and morphological methods.

Methodology	Time cost (day)	Sample limitation	Economical cost (CNY)
DNA metabarcoding	15	Multiple mixed sample	385.6
DNA barcoding	8	Individual	3044.8
Morphological identification	10	Individual	1856

### 3.3 Quality Assessment of DNA Metabarcoding Sequencing Data

Initial sequencing generated 828,704 raw reads (Table S2), of which 828,656 high‐quality reads (99.99% retention rate) were obtained following quality filtering and chimera removal, demonstrating exceptional sequencing quality. Rarefaction analysis revealed an initial rapid increase in OTU accumulation with sequence depth, followed by asymptotic stabilization, indicating sufficient coverage to detect most species present across sampling sites.

### 3.4 Species Composition Across Taxonomic Levels

#### 3.4.1 OTU Clustering Analysis Based on DNA Metabarcoding

Through database queries against the BOLD System and NCBI, we identified 604 OTU sequences, of which 600 OTUs were successfully matched to taxonomic records (453 species, 320 genera, 31 families) while 4 OTUs remained unidentified at the species level (Table 3). Comparative analysis revealed that although forest ecosystems contained more sampling plots than grass‐shrub ecosystems, the latter yielded a greater number of specimens (59.5% of total samples). Among the nine sampling sites, HJV produced the highest specimen count, while HQL yielded the fewest. For Lepidoptera‐specific OTUs, HJV showed low species richness but high abundance, contrasting with HQL which exhibited higher diversity despite low sampling effort. Notably, OTU counts in HQL and GZM slightly exceed their respective physical sample numbers.

**TABLE 3:** Comparative sample and OTU distributions across ecosystem types.

Ecosystem	Sites	No. of specimens	Lepidoptera OTUs	Species identified
Forest	BDG	403	237	216
	XTG	334	225	208
	HQL	173	178	165
	ZGG	375	269	244
	GZM	282	284	247
Grass‐shrub	BSG	374	270	243
	DCP	308	233	214
	KDZ	487	259	237
	HJV	583	216	194
	Total	2944	604	453 (4 unidentified to species)

**FIGURE 2:**
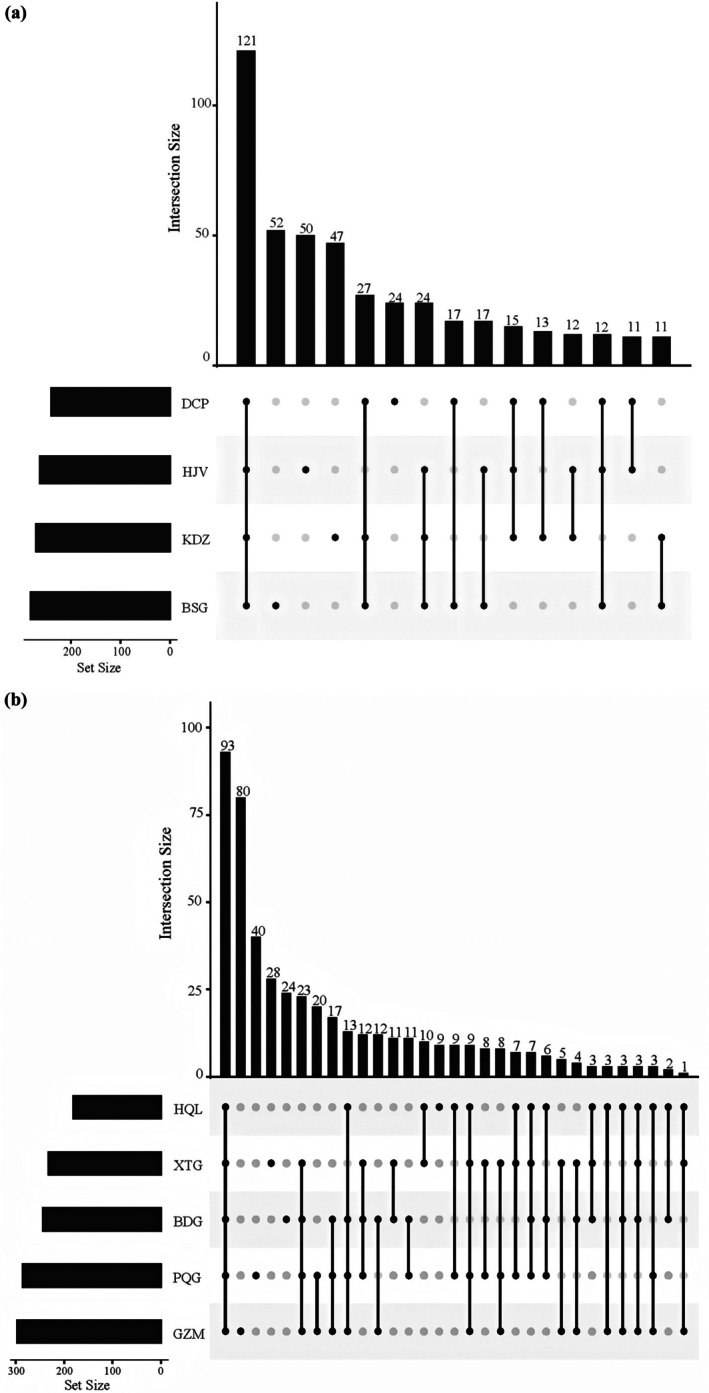
Distribution of shared and unique OTUs in forest and grass‐shrub ecosystems. (a) Shared and unique OTUs in grass‐shrubs. (b) Shared and unique OTUs in forests.

In the grass‐shrub ecosystem, the four sampling sites collectively shared 121 OTUs (Figure 2a). Site‐specific OTU counts reveal distinct diversity patterns: BSG contained 52 unique OTUs, KDZ 47, HJV 50, and DCP 24. These site‐exclusive OTU distributions indicate higher insect diversity at BSG and HJV compared to DCP. The 121 shared OTUs present across all sites demonstrate significant compositional similarity within this ecosystem type.

In the forest ecosystem, the five sampling sites collectively shared 93 OTUs (Figure 2b). Site‐specific OTU composition varied substantially: BDG contained 24 unique OTUs, GZM 80, ZGG 40, HQL 9, and XTG 28. This distribution of unique OTUs demonstrates pronounced diversity differences among sites, with GZM showing the highest richness in biodiversity and HQL the lowest.

#### 3.4.2 Family‐Level Taxonomic Classification Using DNA Metabarcoding

**FIGURE 3:**
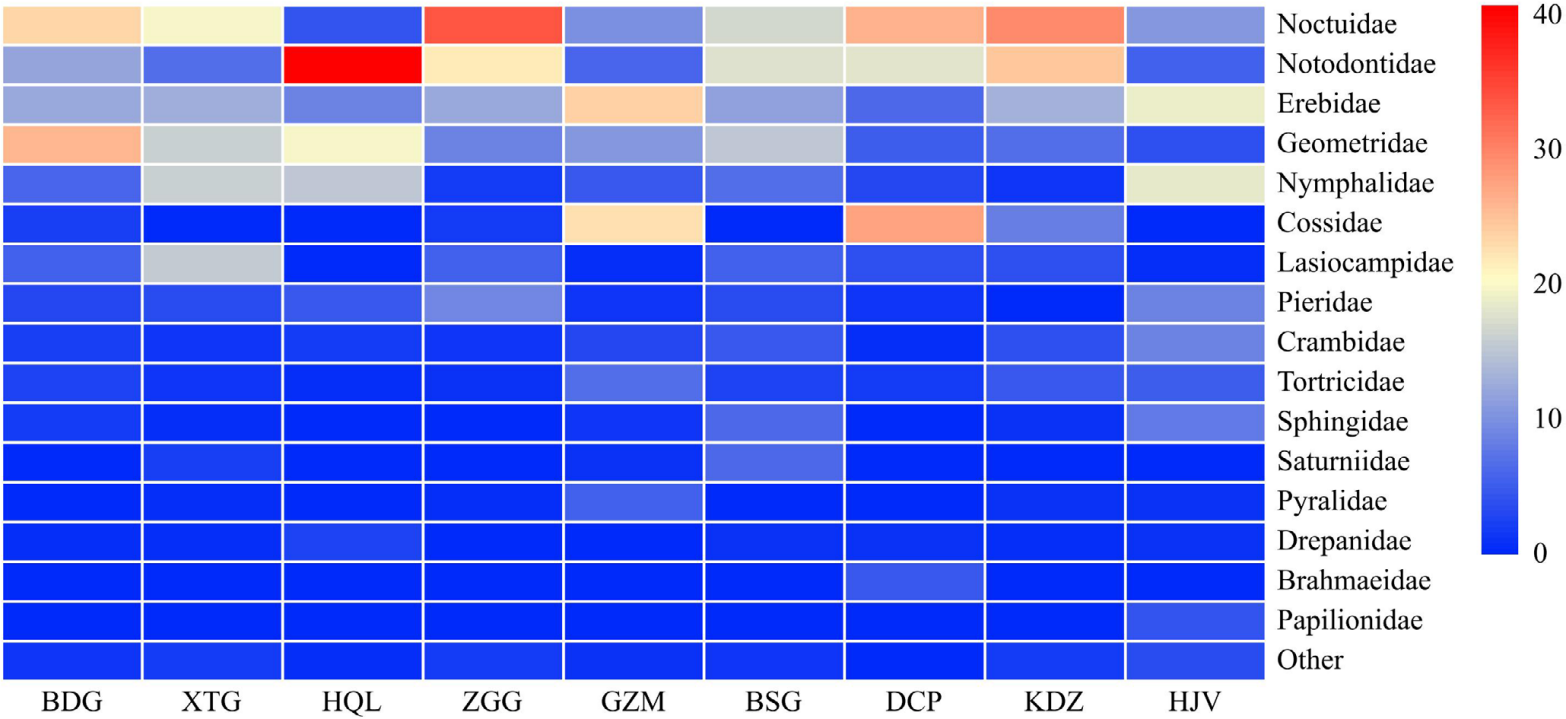
Heatmap of family composition across nine study plots in Pangquangou Families representing < 1% relative abundance across all samples were grouped as “Others”.

Across all nine sampling sites, insect specimens were classified into 31 distinct families (Figure 3). Ecologically, the four grass‐shrub sites each maintained ≥ s10 families, demonstrating more uniform family‐level distribution patterns compared to forest sites. Two dominant families, Noctuidae and Notodontidae, exhibited consistently high relative abundance in both ecosystems. In grass‐shrub habitats, Noctuidae accounted for 21.45% of specimens and Notodontidae for 17.24% whereas in forest habitats, Noctuidae represented 19.07% followed by Notodontidae (16.32%), Geometridae (15.07%), and Erebidae (14.83%).

Family‐level composition analysis across Pangquangou's nine sampling sites (Figure 3) identified five widely distributed and relatively abundant families: Noctuidae, Notodontidae, Geometridae, Cossidae, and Nymphalidae. Notodontidae showed particularly high proportions with HQL (40.78%), followed by KDZ (24.70%) and ZGG (21.95%), with similar proportions observed with DCP (18.08%) and BSG (17.82%). Notably, ZGG displayed the highest relative abundance of Noctuidae (33.47%).

#### 3.4.3 Genus‐Level Taxonomic Profiling Using DNA Metabarcoding

**FIGURE 4:**
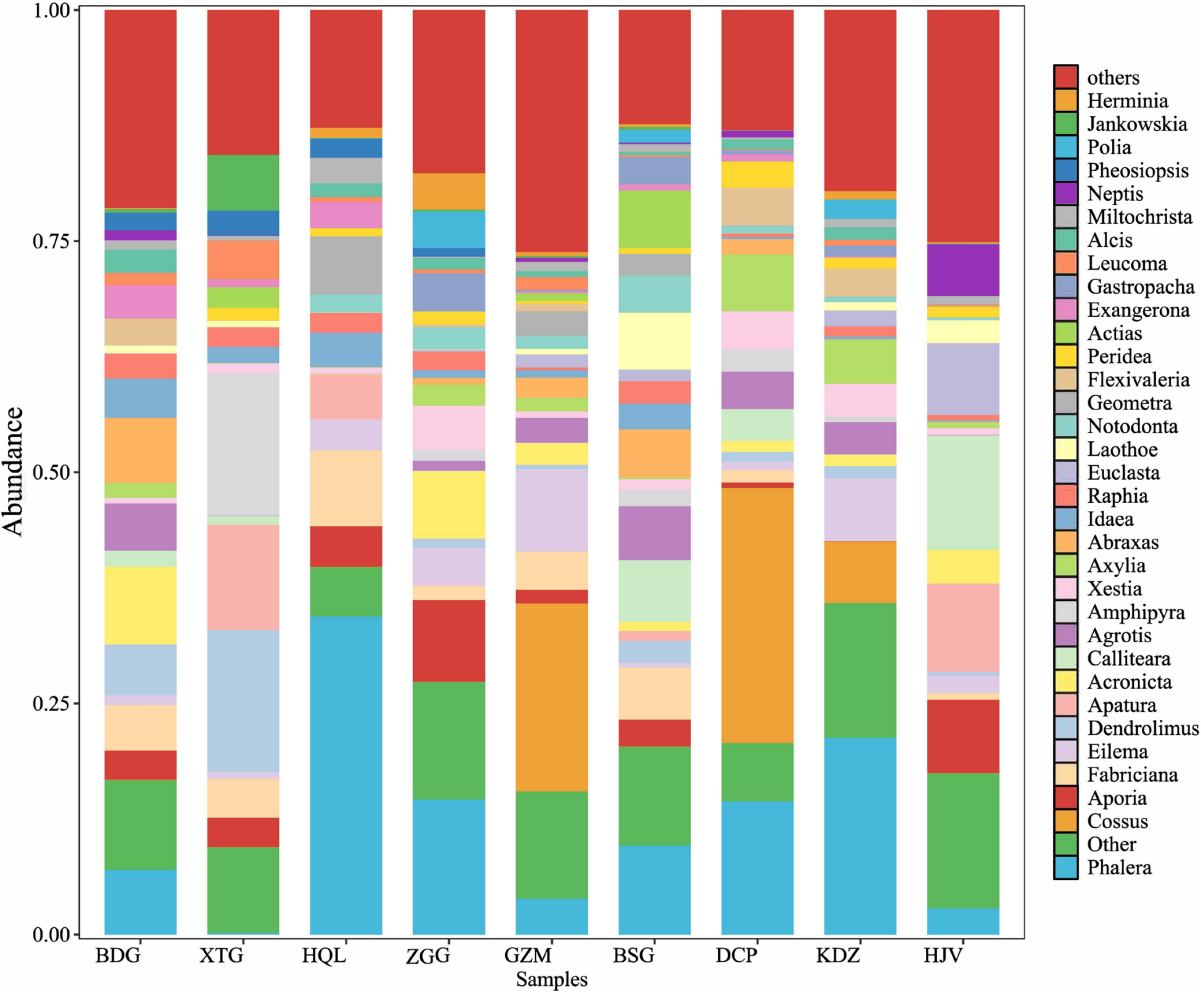
Histogram of genus‐level abundance distribution across study plots.

The 320 genera identified across the nine sampling sites were categorized, with genera showing relative abundance values below 1% in both ecosystems grouped as “Others”. In Pangquangou, *Phalera* and *Cossus* exhibited notably high abundance. In the grass‐shrub ecosystem, *Phalera* accounted for 10.94% of observations, while in the forest ecosystem it represented 12.52%. The abundance of *Cossus* in grass‐shrubs was approximately double that in forests, constituting 9.19% and 5.09% of observations in grass‐shrub and forest ecosystems, respectively (Figure 4).

In the forest ecosystem, the highest relative abundances were observed for *Stenoplastis* in BDG (8.39%), *Amphipyra* in XTG (15.43%), and *Notodonta* in HQL (34.41%), substantially exceeding other genera at these sites. ZGG showed a higher abundance of *Pieris* (8.84%) while GZM was dominated by *Cossus* (20.29%), which was nearly absent in other sites like BDG and XTG (< 1%).

In the grass‐shrub ecosystem, *Phalera* was relatively abundant in both BSG (9.6%) and KDZ (21.26%). *Cossus* showed the highest proportion in DCP (27.57%), nearly doubling the abundance of *Phalera* (14.41%), which ranked second at this site. HJV exhibited notable proportions of *Lymantria* (12.34%) and *Nymphalis* (9.49%), while other genera showed minimal abundance variations (< 5% difference) across these sampling sites.

### 3.5 Alpha‐Diversity Analysis Using DNA Metabarcoding Data

Alpha‐diversity reflects both species richness and evenness within biological communities. Using OTUs clustered from sequence data, we calculated multiple diversity indices (Shannon, Chao1, ACE, Simpson, Shannon Evenness, and Coverage) to assess species richness across sampling sites (Table 4). Among all sites, ZGG showed the highest Shannon diversity index (3.88), followed by BSG (3.85), KDZ (3.84), and BDG (3.83) while HQL exhibited the lowest diversity (Shannon index = 2.94). The Simpson index was highest in HQL and lowest in BSG. All sites maintained Shannon Evenness values exceeding 0.5, with BDG displaying the most uniform species distribution (0.70). Importantly, 100% sequencing coverage was achieved for all sample libraries, ensuring complete sequence detection in this study.

**TABLE 4:** Statistical table of α‐diversity indices across study plots.

Sample	Shannon	Chao	Ace	Simpson	Shannoneven	Coverage
BDG	3.83	305	284	0.035	0.697	1.00
BSG	3.85	313	317	0.036	0.683	1.00
DCP	3.11	309	294	0.111	0.567	1.00
GZM	3.75	316	321	0.061	0.659	1.00
HJV	3.82	277	279	0.044	0.686	1.00
HQL	2.94	217	220	0.137	0.566	1.00
KDZ	3.84	296	294	0.061	0.686	1.00
ZGG	3.88	323	330	0.044	0.686	1.00
XTG	3.39	273	272	0.068	0.623	1.00

**FIGURE 5:**
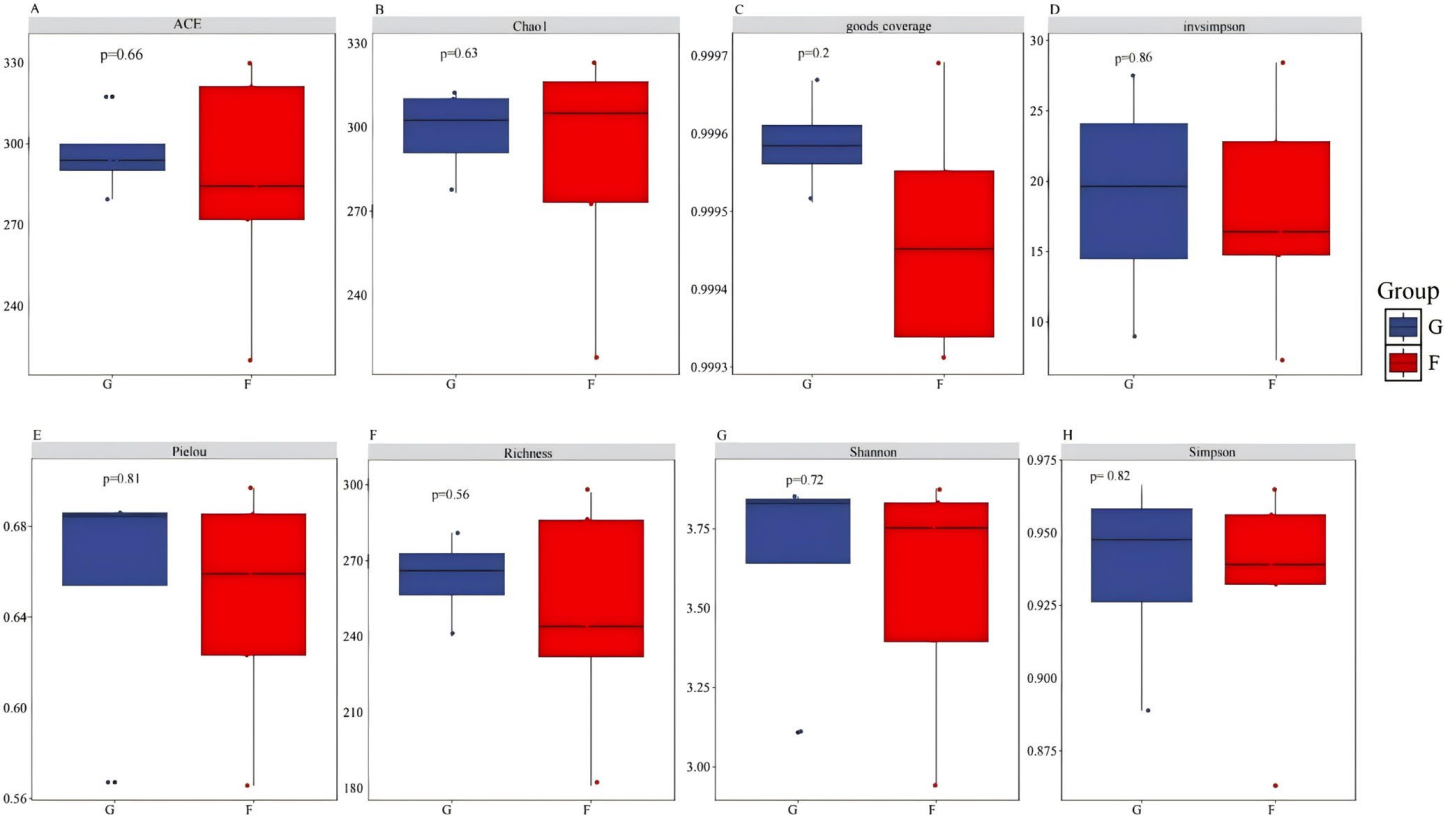
Comparative α‐diversity estimates between the two ecosystems. Boxplots show (a) species richness, (b) Shannon diversity, (c) Simpson index, (d) Pielou's evenness, (e) inverse Simpson index, (f) Chao1 richness estimator, (g) ACE richness estimator, and (h) Good's coverage. Red boxes represent grass‐shrub ecosystems (G) and blue boxes represent forest ecosystems (F).

Comparative analysis of OTU‐based diversity metrics between the two ecosystems revealed no statistically significant differences (Figure 5) across multiple indices: species richness, Shannon diversity index, Simpson diversity index, Pielou evenness index, inverse Simpson index, Chao1 richness estimator, ACE richness estimator, and Goods coverage. The lack of significant differences in these diversity metrics suggests comparable α‐diversity between the two ecosystems.

### 3.6 Beta‐Diversity Analysis Using DNA Metabarcoding Data

**FIGURE 6:**
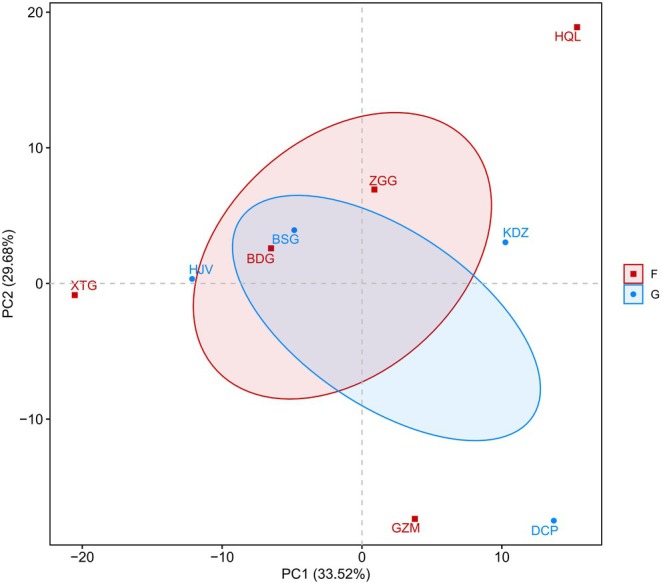
PCA based on two‐ecosystem species composition.

Permutational multivariate analysis of variance (ADONIS) based on Bray‐Curtis distances revealed no significant differences between grass‐shrub and forest ecosystems at any taxonomic level (family: *p* = 0.837; genus: *p* = 0.55; species: *p* = 0.332). Principal component analysis (PCA) of species composition demonstrated both similarities and differences between the two ecosystem types and among plots within each ecosystem. The first two principal components (PC1 and PC2) accounted for 63.2% of the total variation in the Pangquangou dataset. PCA ordination revealed extensive overlap between grass‐shrub and forest ecosystems, indicating high species composition similarity. Notably, the BSG plot (grass‐shrub) clustered most closely with the BDG plot (forest), suggesting particularly similar species assemblages. In contrast, forest ecosystem plots (XTG, GZM, HQL) showed greater dispersion, reflecting distinct species compositions compared to other plots within the same ecosystem. Similarly, within the grass‐shrub ecosystem, the DCP and KDZ plots exhibited clear divergence from other plots in the system (Figure 6).

### 3.7 Distribution in Ecosystems, Ecological Roles, and Types of Damage of Species

A total of 2944 samples representing 453 species were collected and classified based on previous literature and reports (Table S3. List of lepidopteran species sampled with their taxonomic and ecological attributes). The majority (49.89%) of the lepidopteran insects in this study site were associated with forest ecosystems (Table 5). Those belonging to grassland‐forest ecosystems accounted for 21.19%, while those from shrub‐grass ecosystems represented 15.67%. Other ecosystems and unreported species accounted for 5.3% and 7.95%, respectively.

**TABLE 5:** The proportion of species across different ecosystem types.

Type of ecosystems	No. of species	Proportion (%)
Forest ecosystems	226	49.9
Grassland‐forest ecosystems	96	21.2
Grass‐shrub ecosystems	71	15.7
Unknown	36	7.9
Other ecosystems	24	5.3

Based on a comprehensive review of various literature sources, the species identified in this study were categorized into seven types of damage (Table 6): Defoliating pests, Seed and fruit pests, Multidimensional damage pests, Boring pests, Storage pests, Nest parasitic pests, and Unknown. Among these categories, defoliating pests accounted for the highest proportion at 58.72%. The unknown category followed with a significant proportion. The remaining categories—Seed and fruit pests, Multidimensional damage pests, Boring pests, Storage pests, and Nest parasitic pests—accounted for 3.1%, 2.0%, 1.8%, 0.2%, and 0.2%, respectively.

**TABLE 6:** The proportion of species based on types of damage.

Types of damage	No. of species	Proportion (%)
Defoliating pests	387	85.4
Seed and fruit pests	26	5.7
Boring pests	13	2.9
Unknown	13	2.9
Multidimensional damage pests	12	2.6
Nest parasitic pests	1	0.2
Storage pests	1	0.2

However, we also analyzed and statistically categorized the ecological roles of these species within ecosystems (Table 7). A full 98% of the species function as primary consumers. Both secondary consumers and secondary decomposers each account for 0.2%, while only 1.3%, due to undetermined identification, are classified as unknown.

**TABLE 7:** The proportion of species based on ecological role types.

Ecological role	No. of species	Proportion (%)
Unknown	6	1.3
Primary consumer	445	98.2
Primary decomposers	1	0.2
Secondary consumer	1	0.2

## 4 Discussion

This study systematically investigated lepidopteran species composition across Pangquangou's ecosystems by integrating DNA metabarcoding with traditional DNA barcoding and morphological identification. Using light trapping and net sweeping, we collected 2944 specimens from nine sites, identifying 453 species (604 OTUs) across 31 families and 80 genera, with four OTUs remaining unidentified. Results show DNA metabarcoding offers superior identification efficiency (82% consistency with both morphological and traditional barcoding in 50 samples), particularly for large numbers of samples, consistent with previous findings that DNA metabarcoding provides a cost‐ and time‐efficient way to recover information on insect community composition. However, as noted by Li et al. (2023), collection methods crucial for final DNA capture remain both unstandardized and incompletely understood, indicating that poorly understood factors in obtaining bio‐monitoring data may affect results. Notably, in the Heatmap of family composition across nine study plots in Pangquangou, HQL exhibited an anomalously high abundance of Notodontidae, indicating that metabarcoding can precisely capture regional population characteristics. While family‐level composition differed between grass‐shrub and forest ecosystems, α‐ and β‐diversity analyses revealed no significant species‐level differences. This lack of distinction may be attributable to the summer season, during which Lepidoptera are particularly active, potentially introducing bias into the findings (Ruchin 2024). However, summer sampling provides only a snapshot of the transient dispersal dynamics characteristic of adult stages (Yamazaki and Kato 2003). Complementary data from the larval stage are therefore essential, as host plant constraints are considerably more restrictive during this developmental period, potentially accentuating observed differentials. Moreover, frequent species exchange across the forest‐grassland ecotone increases shared species pools.

Additionally, we noted that certain Lepidoptera species, such as those belonging to the Cossidae family, are distributed across both habitats. The prevalence of these species in forest ecosystems, where they constitute a relatively significant proportion in grass‐shrub systems, is hypothesized to be a result of cross‐habitat dispersal (Yakovlev et al. 2016).

Furthermore, the edge effect and the presence of an ecological interlacing zone may facilitate the frequent exchange of matter, energy, and species between the two habitats (Wang et al. 2022). This exchange could lead to a higher proportion of shared species and thereby diminish the observed differences in Lepidoptera distribution between the forest and grass‐shrub ecosystems in this study. Interestingly, in this study, we found that the majority of species in our sampling area have been reported to belong to forest ecosystems in previous studies, accounting for 49.89%, while only 15.67% were reported to belong to shrub‐grass ecosystems. This proportion presents an interesting contrast to the number of samples we collected from forest and shrub‐grass ecosystems: we captured 1567 samples from forest ecosystems, compared to 1752 samples from shrub‐grass ecosystems. This discrepancy may suggest that shrub‐grass ecosystems have a higher potential for species diversity than previously recognized.

In addition, 58.7% of the insects in our study area were identified as defoliating pests. This high proportion is likely related to the vegetation structure of forest and shrub‐grass ecosystems. In the forest communities of Pangquangou Nature Reserve, the richness of the species index of the herb layer is significantly higher than that of the tree and shrub layers (Chen and Zhang 2000). The rich herb layer provides an abundant food resource for defoliating pests, thereby supporting a higher proportion of these pests. Therefore, the herb layer not only contributes the most to the overall diversity of the forest community but also provides an ecological basis for the high proportion of defoliating pests.

During the morphological dissections and traditional barcode identification processes applied to the 50 sampled species, our research may have inadvertently favored species that are well‐documented in existing reference materials. This bias has introduced a systematic bias into our study. Well‐documented species, which have been extensively studied, have accumulated a significant amount of data, while emerging and niche groups still suffer from a lack of basic data, perpetuating a cycle of preferential attention. This limitation is due to the uneven distribution of research resources and the limited access to data for less studied species.

Our results demonstrate the application value of DNA metabarcoding in agricultural pest monitoring and invasive species detection, a conclusion supported by previous studies (Evans et al. 2017; Morales‐Hojas 2017) and consistent with recent reports on its effectiveness for medical entomology (Solano et al. 2011) and biodiversity assessment. Furthermore, this study offers new insights into biodiversity distribution patterns.

The scientific significance of this work lies in establishing an efficient, standardized lepidopteran identification system for biodiversity monitoring and pest management. As exemplified by Cossidae (wood‐boring) pests in this study, DNA metabarcoding may enable early detection of infestations (Qi et al. 2025), potentially identifying invasive stages 2–3 developmental phases ahead of conventional trapping methods. This stuy was operationalized by establishing, for the first time in the Pangquangou Nature Reserve, a “morphology‐barcode‐metabarcoding” tripartite monitoring protocol, which can serve as a standardized template for other protected areas.

Practically, metabarcoding's high‐throughput nature makes it ideal for training protected area staff in biodiversity monitoring. For Pangquangou, we recommend bi‐monthly sampling across the forest‐grassland ecotone during spring and autumn to capture both adult and larval stages, thereby reducing seasonal bias and improving early warning accuracy for pests like Cossidae.

## 5 Conclusion

This study revealed high insect community diversity in Pangquangou National Nature Reserve using DNA metabarcoding, detecting 604 OTUs encompassing 453 species. No significant differences were observed in species richness or community composition between forest and grass‐shrub ecosystems, though grass‐shrub habitats contributed 59.5% of the total specimens. Noctuidae and Notodontidae were the dominant families in both ecosystems, indicating similar ecological functional structures. Methodological comparison confirmed that DNA metabarcoding maintains 82% identification accuracy while reducing costs by 79.2% compared to morphological identification. However, variation in species composition among sampling sites suggests that collection methods significantly influence results. Ecological function analysis revealed that primary consumers dominated the community (98%), with defoliating pests representing the primary damage type. These findings underscore the necessity of standardizing sampling strategies when applying metabarcoding techniques. Future research should explore the relationship between sampling methods and DNA capture efficiency to enhance the reliability of biodiversity monitoring.

## Author Contributions

**Ling Wu:** data curation (equal), formal analysis (equal), investigation (equal), writing – original draft (equal), writing – review and editing (equal). **Ziyu Huang:** formal analysis (equal), investigation (equal), visualization (equal), writing – original draft (equal). **Xinrun Ren:** data curation (equal), formal analysis (equal), writing – original draft (equal). **Huimin Yao:** formal analysis (equal), investigation (equal), writing – original draft (equal). **Min Li:** formal analysis (equal), investigation (equal), visualization (equal), writing – original draft (equal). **Angie Deng:** formal analysis (equal), investigation (equal), writing – original draft (equal), writing – review and editing (equal). **Yali Li:** investigation (equal). **Shiyi Lian:** investigation (equal). **Lina Men:** formal analysis (equal), methodology (equal), supervision (equal), writing – original draft (equal), writing – review and editing (equal). **Zhiwei Zhang:** funding acquisition (equal), project administration (equal), resources (equal), supervision (equal), visualization (equal), writing – review and editing (equal).

## Funding

This work was supported by National Natural Science Foundation of China (Grant 32171806), Financial Funds of Shanxi Province, Shanxi Dangerous Forest & Grassland Pest Inspection, Undergraduate Innovation Project of ShanXi Province (Grant S202410113058), and Graduate Student Innovation Project of Shanxi Province (Grant 2025SJ199).

## Conflicts of Interest

The authors declare no conflicts of interest.

## Supporting information


**Table S1:** A comparative validation of species identification efficacy: DNA barcoding, metabarcoding, and morphological approaches.

**Table S2:** Raw data.

**Table S3:** List of lepidopteran species sampled with their taxonomic and ecological attributes.

## Data Availability

The raw sequencing data supporting the results of this study have been deposited in the Genome Sequence Archive (GSA) of the China National Center for Bioinformation (CNCB) under accession number PRJCA063522. Further inquiries can be directed to the corresponding authors.
